# A retrospective record review and assessment of cost of quetiapine use in a community psychiatric setting in the Sedibeng district of Gauteng

**DOI:** 10.4102/sajpsychiatry.v23i0.1057

**Published:** 2017-07-17

**Authors:** Lesley J. Robertson, Jacqui K. Miot, Bernard Janse van Rensburg

**Affiliations:** 1 Department of Psychiatry, School of Clinical Medicine, University of the Witwatersrand, South Africa; 2 Department of Pharmacy and Pharmacology, Faculty of Health Sciences, University of the Witwatersrand, South Africa

## Abstract

**Background:**

With the revision of the National Essential Medicines List in South Africa, quetiapine is only available at the discretion of individual institutions in the public health sector. However, quetiapine is effective in managing all aspects of bipolar disorder, including preventative treatment of depressive episodes, and may be a cost-effective option in severe illness.

**Aim:**

To present the first retrospective review of quetiapine use in a peri-urban health district of South Africa, describing the patient profile, clinical response and prescribing patterns.

**Methods:**

The clinical files of all patients in Sedibeng District who received quetiapine over a defined 3-year period (2011–2013) were reviewed. A positive clinical response was defined as both symptomatic and functional improvement. Demographic and clinical characteristics of responders were compared with that of non-responders. Pre- and post-quetiapine scripts of the responders were audited and costed.

**Results:**

Patients who received quetiapine (*n* = 40) had chronic disabling illness, often with multiple medication trials and hospitalisations prior to quetiapine use. Bipolar II disorder (followed by bipolar I disorder) was the most common primary psychiatric diagnosis documented. Other than improvement in functioning (*p* < 0.0001), responders differed significantly from non-responders in terms of a higher level of polypharmacy and a significant reduction in median number of medications from pre- to post-quetiapine (*p* = 0.0057).

**Conclusion:**

Quetiapine use was associated with a highly significant improvement in functioning; however, it came at a 52% increase in medicine cost. Pre-quetiapine treatments, though, did not achieve an optimal level of functioning, and overall costs may be reduced by more rational prescribing habits.

## Introduction

Cost awareness on the part of doctors is essential in Africa with its scarce resources and high disease burden, yet medical practitioners rarely audit the cost of the treatment they prescribe.^1^ Nevertheless, such information may assist in the development of an affordable, clinically appropriate Essential Medicines List (EML). For the management of bipolar disorder (BD) in low- and middle-income countries (LMICs), only lithium, carbamazepine and valproate are listed on the EML of the World Health Organization (WHO).^2^ There is only one second-generation antipsychotic (SGA), risperidone, specifically for psychotic disorders.

However, with the availability of generic medication, an argument has been made for the wider use of SGAs in LMICs in the treatment of BD.^3^ One such SGA is quetiapine, which has been approved by the US Food and Drug Administration for use in the acute and maintenance treatment of manic and depressive episodes in BD, as well as in schizophrenia and major depressive disorder (MDD).^4^

In South Africa, with the revision of the National Essential Medicines List (NEML) to allow for universal health coverage,^5^ most SGAs have come under review. Quetiapine is not on the NEML,^6^ despite it being the only first-line treatment recommended for acute bipolar II depression in the South African Society of Psychiatrists (SASOP)’s treatment guidelines.^7^ Lithium, lamotrigine and the combination of olanzapine and fluoxetine constitute the medications on the South African NEML with proven efficacy in the treatment of bipolar depression.^6^ Lithium is arguably the gold standard in the long-term treatment of BD.^8^ However, its delayed antidepressant effect makes it less efficacious in acute treatment, and the risk of adverse effects may limit its usefulness. Lamotrigine is recommended only as adjunctive maintenance treatment. While there is good evidence for the addition of fluoxetine to olanzapine in acute bipolar depression,^9^ evidence is weak for either the combination or for olanzapine alone in the prevention of relapse into depression.^10^

In the management of BD, the range of efficacious medicines for the treatment of depressive episodes is considerably more limited than that for mania.^9,10^ In addition, medication effective in acute bipolar depression (e.g. the addition of an antidepressant to a mood stabiliser) may not be effective in the prevention of relapse into a depressive episode. Furthermore, depressive episodes confer severe long-term disability and carry the highest suicide risk of all psychiatric disorders.^9^ Depressive episodes are more common in women with BD than in men, often occur during the reproductive years, persist into later life,^11^ and, as with any severe maternal depressive disorder, may have negative effects on the mental and physical health of their offspring.^12^

Although not on the NEML, quetiapine is currently available in the public health sector^13^ at the discretion of individual institutions and clinician motivation. The strongest evidence for its use over other SGAs on the NEML is in the management of BD, particularly depressive episodes, rather than the management of schizophrenia.^14^ In terms of efficacy, quetiapine is equivalent to the olanzapine–fluoxetine combination in acute bipolar depression^9^ and to lithium in long-term treatment,^8,10^ but, at 2015 government tender prices, it was more expensive than either.^13^

## Aim

The aim of this study was to evaluate the use of quetiapine over a 3-year period in the community psychiatric clinics of the Sedibeng district of Gauteng, where the extended-release (XR) form of quetiapine has been available.

The first objective was to describe the patient population for whom quetiapine XR was prescribed, to describe the clinical response; and to compare those who responded to quetiapine (responders) with those who did not (non-responders) in terms of demographic and clinical profile, polypharmacy and side-effect burden. The second objective was to make an assessment of the direct pharmaceutical cost implications for those in whom a positive clinical response to quetiapine was evident in the clinical records. The third objective was to ascertain whether the use of quetiapine was judicious according to approved indications, current evidence and treatment guidelines.^4,7,9,10,14^

## Methods

### Study design and setting

A retrospective record review of all patients who received quetiapine XR in the Sedibeng district psychiatry clinics between January 2011 and December 2013 was performed. The clinical records were examined up to the end of July 2014 in order to allow time for a sustained clinical response.

Sedibeng is a peri-urban district in Southern Gauteng with a total population of 916 484 as of 2011.^15^ Its public health sector comprises eight psychiatric clinics which are linked to the University of the Witwatersrand. These clinics are staffed mainly by psychiatric registrars, who provide clinical service on a 6 monthly basis during the course of their registrar rotation, as well as the Sedibeng district psychiatric nurses. On average, these clinics served just under 3800 adults with mental illness during the study period, which is roughly 0.5% of the district’s total adult population.^16^ There are no published data regarding the prevalence of mental illness in the district, the profile of disorders treated within the psychiatric clinics or the prescribing patterns of psychotropic medication.

### Study population

All patients for whom quetiapine was prescribed between January 2011 and December 2013 were included in the study. Those in whom a positive clinical response was evident from the clinical records were included in the pre- and post-quetiapine costing assessment.

### Data collection

A list of patients who were prescribed quetiapine during the study period was obtained from the district pharmacy. Clinic registers were also checked to ensure that no patients were missed. The clinical records of each patient were then examined for demographic details, response to quetiapine, clinical diagnosis and prescribing patterns.

The clinical response to quetiapine was ascertained retrospectively from clinical notes and nursing records. As rating scales are not used routinely in the district psychiatric clinics, one had to be applied retrospectively in order to assess the response to treatment in an objective and quantifiable manner. The Global Assessment of Functioning (GAF) Scale was used as clinical records are a recognised source of information for the GAF Scale, it is simple, it provides an overall measure of both symptoms and impairment across all psychiatric disorders, and it may be used to reflect a change in the patient’s condition over time.^17,18^

Although the two dimensions of the scale have been shown to be valid in terms of reflecting both symptomatic distress and social functioning, its reliability has been questioned, especially in the routine clinical setting with different raters of varying experience using different sources of information for the same patient. Although it may be more reliable when applied retrospectively in an objective manner by one investigator, its validity is dependent on the quality and accuracy of the available notes. Therefore, all clinical notes, including those made by the nursing staff, social workers, allied professionals and referring doctors, were used to determine which of the 10 anchor points was most applicable prior to quetiapine initiation (pre-quetiapine) and at July 2014 or at quetiapine discontinuation if this had occurred (post-quetiapine). A positive clinical response was defined as an improvement of at least one decile in the GAF Scale, taking the lower of the two dimensions as the reference point for the pre- and post-quetiapine measures.

For those with a positive clinical response, the pre- and post-quetiapine prescriptions were costed. The costing analysis was performed on the script immediately before quetiapine was prescribed, being the treatment which was deemed clinically necessary to change because of poor response and/or adverse effects, and the last script on quetiapine before the analysis ended, being the medication which had resulted in improved functioning. For consistency in cost comparison, the average monthly cost per medicine and per script was calculated using prices from the national procurement catalogue of June 2015, being the prices at the time of the analysis. Only medicine costs were determined. Depending on dosage, costs of quetiapine XR 50 mg, 200 mg, 300 mg and 400 mg were used. Scripts for non-responders were not included in the costing analysis as quetiapine would have been stopped and alternative clinical decisions would have been made.

### Statistical analysis

Data analysis was carried out using Statistical Analysis Software (SAS) Version 9.3 for Windows. The 5% significance level (*p* < 0.05) was used throughout. Descriptive analysis of the data was carried out for categorical variables using frequency and percentage tabulation, and for continuous variables using the mean, standard deviation, median and interquartile range. The association between categorical study variables and responder or non-responder groups was determined by the chi-squared test (Fisher’s exact test was used for 2 x 2 tables or where the requirements for the chi-squared test could not be met). The relationship between continuous study variables and responder/non-responder group was assessed by the *t*-test. Where data did not meet the assumptions of the *t*-test, a non-parametric alternative, the Wilcoxon rank sum test, was used. The relationship between GAF score categories before and after the study was assessed by the Stuart-Maxwell test, while the relationship between the pill count before and after the study was assessed by the Wilcoxon matched-pairs test.

## Ethical consideration

Ethics approval was granted by the Human Research Ethics Committee of the University of the Witwatersrand.

## Results

### Demographic profile

Only 40 patients in the Sedibeng district psychiatric clinics received quetiapine over the 3 years. Demographic data are presented in Table 1. All 40 patients were adults; the mean age at the time of record review was 45 years (SD 12 years; range 18–65 years) and the median age of onset of illness was 27 years (interquartile range [IQR)] 21–38 years; range 12–63 years). The median duration of illness was 13.5 years (IQR 6–19 years; range 0–41 years), with a median of 11 years (IQR 4–20 years; range 0–39 years) prior to quetiapine use.

**TABLE 1:** Demographic profile of the overall group (*n* = 40).

Variable	*n*	%
**Sex**		
Female	32	80.0
Male	8	20.0
**Ethnicity**		
Black	22	55.0
White	15	37.5
Indian	2	5.0
Coloured†	1	2.5
**Relationship status**		
Single	27	67.5
In a relationship	13	32.5
**Employment status**		
Unemployed	25	62.5
Employed	11	27.5
Student (tertiary institution)	3	7.5
Scholar	1	2.5
**HLOE**		
Primary	1	2.5
Secondary	13	32.5
Matric	19	47.5
Tertiary	7	17.5

† , the South African national census uses the term ‘Coloured’, instead of Mixed race.

HLOE, highest level of education.

### Clinical response

**FIGURE 1:**
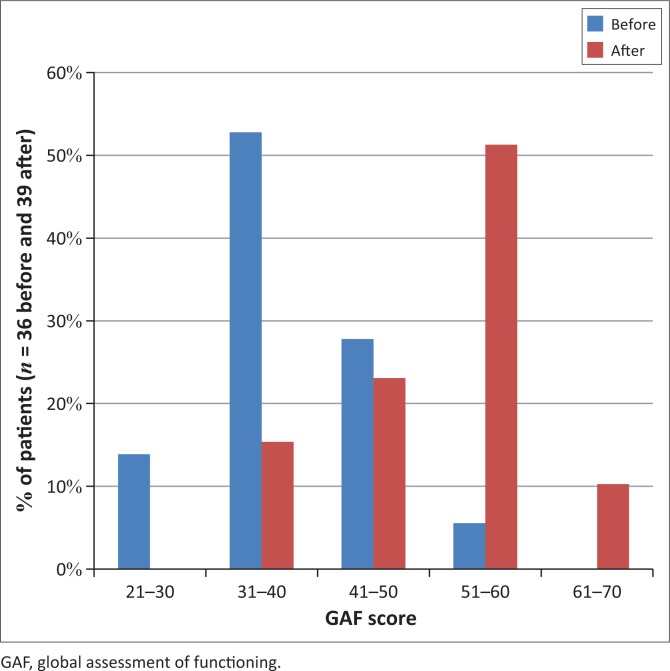
Global assessment of functioning score before (*n* = 36) and after (*n* = 39) initiation of quetiapine.

The pre- and post-quetiapine GAF scores of the sample are illustrated in Figure 1. In four patients, a pre-quetiapine GAF score could not be assigned as the quetiapine had been initiated in the private sector and insufficient detail as to their initial presentation and functioning was available in the clinic records. However, a clear history of a positive response was documented in the notes with the need to continue quetiapine in three of the four patients, one of whom also had details of the pre-quetiapine script. In one of these three, the post-quetiapine score could not be evaluated as the patient had returned to the private sector prior to July 2014 and there was insufficient information in the records as to their symptoms or social functioning. Regarding the fourth patient, with a diagnosis of traumatic brain injury (TBI) and epilepsy, the notes indicated a poor response (GAF score = 41–50), quetiapine was discontinued and anticonvulsant medication was optimised.

Of the 40 patients, 13 (32%) were grouped as non-responders and 27 (68%) as responders (including the three referred from the private sector mentioned above). Four patients were designated non-responders despite improvement on the functional dimension of the GAF scale. Two of these patients had persistent distressing psychotic symptoms resulting in a lower overall GAF score despite an improvement in mood and level of function. In the other two, the better functioning was deemed from the records to be because of psychosocial interventions rather than medication, and quetiapine use had been stopped in both. Although this could be considered to be inconsistent with the criteria, as the overall GAF score had improved, they could not be included as responders as evidence in the notes revealed otherwise. One patient (a 63-year-old with dementia) was grouped as a non-responder despite a symptomatic response as there was no improvement on the functional dimension.

Of those in whom the GAF score was ascertained, 95% of patients were seriously ill and/or functionally impaired with scores of 41–50 and below prior to quetiapine use. Post-quetiapine use, 61% of patients had a GAF score of 51–60 or above, a level of functioning at which an individual may sustain employment or care for children. The GAF score improved significantly overall and for the responder group (*p* < 0.0001), with significantly higher final scores for responders than non-responders (*p* = 0.0002; phi coefficient = 0.68). As expected, there was no overall improvement in GAF score for non-responders (*p* = 0.52).

There were no significant differences between responders and non-responders regarding the demographic profile, age of onset, duration of illness, pre-quetiapine GAF score or number of hospitalisations. A history of repeated hospitalisation was common, with about half (51%) of the 40 patients having had three or more admissions and one-third of the patients having five or more admissions before initiation on quetiapine. After quetiapine use, over a time range of 5–48 months, there was one admission amongst the responders, which was related to HIV infection, and three amongst the non-responders. Because of the disparate time periods it was not possible to do a before and after comparison for hospitalisations.

### Clinical profile

Primary and co-morbid diagnoses are summarised in Table 2. There were no significant differences between responders and non-responders. However, BD I and BD II occurred more frequently amongst responders and MDD was more common amongst non-responders, with a trend towards significance for BD II and MDD (*p* = 0.06 and 0.07, respectively). Clinical features of psychotic depression were evident in the notes of 22 patients (38% of non-responders and 63% of responders; *p* = 0.19). Although not a diagnostic category, these features have been included in Table 2 as a point of clinical interest. In three patients, the psychiatric symptoms were deemed to be secondary to another medical condition, namely HIV infection, TBI with epilepsy and dementia due to multiple aetiologies. Comorbid medical illness was common in both groups.

**TABLE 2:** Primary and comorbid diagnoses.

Category	Overall *n* = 40		Non-responders *n* = 13		Responders *n* = 27	
		
	*N*	%	*n*	%	*N*	%
**Primary psychiatric diagnosis**						
BD II	12	30	1	8	11	41
BD I	11	28	2	15	9	33
MDD	6	15	4	31	2	7
Schizoaffective disorder	6	15	2	15	4	15
Mood and/or psychosis due to AMC	3	8	2	15	1	4
Schizophrenia	1	3	1	8	0	0
None (V code: Malingering)	1	3	1	8	0	0
**Comorbid psychiatric diagnoses**						
Clinical features of psychotic depression	22	55	5	38	17	63
GAD	8	20	3	23	5	19
PTSD	2	5	1	8	1	4
Social anxiety disorder	1	3	0	0	1	4
OCD	1	3	0	0	1	4
Benzodiazepine use disorder	5	13	3	23	2	7
Cannabis use disorder	3	8	2	15	1	4
Alcohol use disorder	1	3	0	0	1	4
Cocaine use disorder	1	3	1	8	0	0
Heroin use disorder	1	3	0	0	1	4
**V codes**						
Bereavement	3	8	2	15	1	4
Malingering	1	3	1	8	0	0
**Traits of personality disorder**						
Cluster B	10	25	4	31	6	22
Cluster C	9	23	4	31	5	19
**Medical co-morbidity**						
Hypertension	5	13	0	0	5	19
Hypercholesterolaemia	4	10	1	8	3	11
Hypothyroidism	4	10	1	8	3	11
HIV	3	8	0	0	3	11
Peptic ulcer disease	2	5	0	0	2	7
Ulcerative colitis	2	5	0	0	2	7
Cardiac failure	1	3	1	8	0	0
Dementia	1	3	1	8	0	0
Diabetes mellitus	1	3	0	0	1	4
Epilepsy – idiopathic	1	3	0	0	1	4
Neurocysticercosis with epilepsy	1	3	0	0	1	4
TBI with epilepsy	1	3	1	8	0	0

BD II, bipolar II disorder; BD I, bipolar I disorder; MDD, major depressive disorder; AMC, another medical condition; GAD, generalised anxiety disorder; PTSD, post-traumatic stress disorder; OCD, obsessive compulsive disorder; HIV, human immunodeficiency virus; TBI, traumatic brain injury.

### Prescribing patterns

All but two patients were initiated on quetiapine as outpatients, during maintenance treatment. Most had previously received multiple trials of medication (Table 3), with no significant difference between responders and non-responders. About a quarter had received three or more trials of different antipsychotics and another quarter received three or more trials of antidepressants. Of the mood stabilisers, valproate and lamotrigine were used most often, with only 38% of patients having had a trial of lithium.

**TABLE 3:** Percentage of patients who had received previous medication trials.

Medication	Overall *n* = 40		Non-responders *n* = 13		Responders *n* = 27	
		
	*N*	%	*n*	%	*N*	%
Antipsychotic	33	83	13	100	20	74
≥ 3 trials of different antipsychotics	9	23	5	38	4	15
Risperidone	32	80	13	100	19	70
Haloperidol/typical LAI	13	33	7	54	6	22
Olanzapine	12	30	2	15	10	37
Clozapine	4	10	2	15	2	7
Chlorpromazine	2	5	0	0	2	7
Sulpiride	1	3	0	0	1	4
Antidepressant	31	78	11	85	20	74
≥ 3 trials of different antidepressants	10	25	2	15	8	30
SSRI	30	75	10	77	20	74
Amitriptyline	11	28	3	23	8	30
SNRI (venlafaxine)	9	23	2	15	7	26
Anticonvulsant	28	70	9	69	19	70
≥ 3 trials of different anticonvulsants	2	5	0	0	2	7
Valproate	19	48	5	38	14	52
Lamotrigine	18	45	5	38	13	48
Carbamazepine	4	10	1	8	3	11
Gabapentin	1	3	0	0	1	4
Lithium (missing: *n* = 2)	15	38	3	23	12	44
ECT	3	8	0	0	3	11
Missing data	3	8	0	0	3	11

LAI, long acting injectable; SSRI, selective serotonin reuptake inhibitor; SNRI, serotonin noradrenaline reuptake inhibitor; ECT =, electroconvulsive therapy.

#### Quetiapine use

The median quetiapine dose was 300 mg (IQR 200 mg–400 mg; range 50 mg–800 mg), with no significant difference in dose between responders and non-responders (*p* = 0.34). Three patients were identified as possibly not warranting a clinical trial: one patient with dementia and two others in whom quetiapine 50 mg nocte was prescribed simultaneously with a selective serotonin reuptake inhibitor (SSRI) at the initial presentation, which was primarily for a depressed mood.

For responders, the mean duration of quetiapine use, and duration of improved function, was 23 months (range of 5–48 months, s.d. = 11). None of the responders discontinued quetiapine during the study period, apart from one patient who stopped treatment at only 5 months, after having started a new job. This appeared to be related to stigma and insufficient psychosocial support. The duration of quetiapine prescriptions in non-responders was significantly lower (*p* = 0.0001) with a mean of 9 months (range of 1–19 months, s.d. = 5).

#### Adverse effects

**FIGURE 2:**
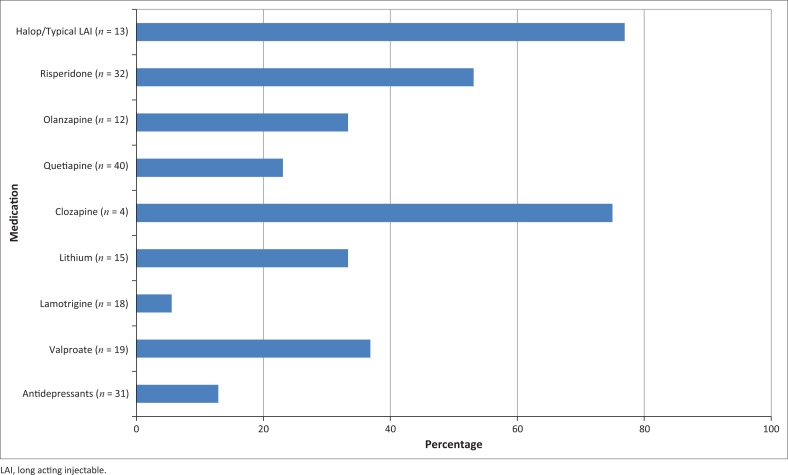
Percentage of patients on a medication who reported side effects.

Adverse effects were noted most frequently with typical antipsychotics and least commonly with antidepressants and lamotrigine (Figure 2), although significance could not be determined because of the small sample size for these medications. Side effects of quetiapine use were significantly more common amongst non-responders than amongst responders (46% and 12% respectively; *p* = 0.039) and consisted of postural hypotension, weight gain, sedation and daytime lethargy. The effects of quetiapine on weight, blood pressure, cholesterol and glycaemic control were not explored with comparison to other medications. Diligence in clinical monitoring was poor: cholesterol and glucose were only monitored in 12% of patients, and weight and blood pressure were monitored in 30% of patients.

#### Polypharmacy

Evaluated as the number of different psychotropic and related medications per day, excluding medications for comorbid medical illness, polypharmacy was greater amongst responders than amongst non-responders (*p* = 0.045 pre-quetiapine; *p* = 0.046 post-quetiapine). Responders were prescribed a mean of 4.0 types of medication (range of 1–9, s.d. = 2.0) per patient pre-quetiapine and 2.9 (range of 1–5, s.d. = 1.1) post-quetiapine, whereas non-responders received 2.7 (range of 1–4, s.d. = 1.1) and 2.2 (range 0–3, s.d. = 1.0) types of medication pre- and post-quetiapine, respectively. The drop in the median number of scripts from pre- to post-quetiapine was significant (*p* = 0.0057) for responders but not for non-responders (*p* = 0.20) (Table 4).

**TABLE 4:** Average pre-and post-quetiapine medication cost per patient (responder).

Medication	Pre-quetiapine *n* = 25 (missing: *n* = 2)		Post-quetiapine *n* = 25 (excluded: *n* = 2 with missing pre-quetiapine data)	
	
	Number of scripts	Average cost/script/month	Number of scripts	Average cost/script/month
Quetiapine	0		25	R119.58
Haloperidol/Typical LAI	3	R21.32	0	
Risperidone	10	R10.96	1	R9.42
Olanzapine	3	R50.16	0	
Chlorpromazine	1	R20.40	0	
SSRI	14	R9.42	6	R6.95
SNRI (venlafaxine)	3	R133.41	2	R108.32
TCA	7	R5.45	3	R6.30
Valproate	10	R63.39	6	R72.41
Lamotrigine	5	R23.90	10	R22.01
Carbamazepine	1	R25.18	0	
Lithium	9	R55.70	4	R66.87
Benzodiazepine	13	R47.72	9	R85.32
Thyroxine	2	R5.35	4	R4.51
Other	16	R43.22	1	R25.65

**Average cost per patient per month**		**R132.00 (s.d. 107.50)**		**R200.37 (s.d. 98.70)**

LAI, long acting injectable; SNRI, serotonin noradrenaline reuptake inhibitor; SSRI, selective serotonin reuptake inhibitor; TCA, tricyclic antidepressant; s.d., standard deviation.

#### Benzodiazepines

As quetiapine is both sedating and anxiolytic, reduced use of benzodiazepines had been anticipated but did not materialise. The number of benzodiazepine scripts decreased insignificantly from 13 to 9, but the average cost per patient almost doubled. The cost increase post-quetiapine was because of an increased dosage in four prescriptions and a reduced dosage to the more expensive 0.5 mg tablets of clonazepam in only two scripts. Of note, two patients were initiated on benzodiazepines simultaneously with the quetiapine prescription. Hence, of the original 13 scripts, the benzodiazepine was discontinued in 6 patients but commenced in 2 others.

#### Cost

The cost implications of using quetiapine to achieve the improvement in GAF score amongst those who responded are summarised in Table 4. The average medication costs per patient for 1 month increased by 52%, despite a reduction in concomitant medicines (other than lamotrigine and thyroxine, which both doubled in use). Apart from quetiapine, valproate and benzodiazepine are the greatest cost drivers because of frequency of prescription. Venlafaxine, the most expensive per script, was reduced in dosage and in number of scripts post-quetiapine. Although inexpensive, it is noteworthy from a clinical perspective that other antidepressants were also reduced, from 21 to 9 scripts.

## Discussion

Given that BD affects at least 1% of the general adult population,^11^ the sample of 40 patients, out of approximately 3800 adults with mental illness attending the clinics, reflects a very low prevalence of quetiapine use in this setting. This is possibly related to its non-availability in the non-academic regional hospital psychiatric unit, but may also be because of poor clinical recognition of bipolar depression with persistent use of other psychotropic medication. The demographic and clinical characteristics of the sample are consistent with those of bipolar depression,^11^ being predominantly female with onset of the disorder during the mid-20s, frequent medical comorbidity and a chronic, disabling course of severe illness with multiple hospitalisations and numerous trials of medication.

Overall, quetiapine use appears to have been judicious according to approved indications and treatment guidelines, a working differential diagnosis of bipolar depression and a median dose of 300 mg, the recommended dose for prevention of relapse into a depressive episode in BD.^4,7,8,10^ However, the average duration of 9 months for non-responders could have been shortened with careful clinical follow-up. Of greater concern, however, is the possible much larger number of unrecognised patients in the clinics and in the district.

Responders differed significantly from non-responders only in polypharmacy, both pre- and post- quetiapine. Although possible clinical reasons for the greater polypharmacy could not be deduced from this study, it may reflect difficulty in managing BD, which accounted for 74% of primary diagnoses in the responder group. The use of risperidone, typical antipsychotics and antidepressants instead of mood stabilisers or antipsychotics with mood stabilising properties is consistent with the literature, which describes ongoing use of these medications in response to psychotic and depressive symptoms in bipolar depression.^7,9,10^ The overall prescription change, with replacement of risperidone and olanzapine with quetiapine and the doubling in scripts of lamotrigine, is consistent with a South Korean observational study on the transition from acute to maintenance treatment in BD.^19^ The increase in quetiapine use is probably related to its efficacy in treating the emergence of depressive symptoms during maintenance care.

The use of benzodiazepines prior to quetiapine may reflect persistent anxiety and insomnia despite existing pharmacotherapy. However, long-term use is associated with serious morbidity and dependency.^20^ As long-term benzodiazepine use shows no benefit in BD,^21^ the ongoing use in the face of clinical improvement is worrisome. That the clinician may play a role in the perpetuation of benzodiazepine addiction has been documented.^20^ The possibility exists in this sample, as doses were increased in four of the responders, whereas the other three substance use disorders (alcohol, cannabis and heroine) in the group all improved with clinical improvement.

Although the 52% cost increase appears high for South Africa, the pre-quetiapine average cost of R132.00 per patient per month achieved a level of functioning in most patients below that required to attend to a child or keep a job, accompanied by residual psychiatric symptoms and a higher pill burden. An increase of R68.37 per patient per month may well be justified through improved clinical outcomes and reduced utilisation of the healthcare system, as has been shown in international health economics analyses.^22^ However, we may not be ‘doing the best with what we have’.^5^ Earlier and more frequent prescription of lithium and/or lamotrigine could possibly reduce the number of antidepressant and antipsychotic scripts and improve clinical outcomes,^8,23^ although their use may be limited by adverse effects.

### Limitations

A major limiting factor of this paper is the retrospective study design and dependence on routine clinical records, which may have affected the reliability of the findings. Regarding the GAF Scale, some subjectivity in allocation of a score is unavoidable. In addition, a disadvantage of only using the lower score of the two dimensions, as opposed to both a symptom and a function score, may have caused some responders (or partial responders) to have been labelled as non-responders. Regarding the previous trials of medication, the study design precluded accurate analysis of the adequacy of each trial. Finally, to give a true reflection of expenses, the costing exercise could have included the cost of quetiapine use in non-responders as ‘wasted resources’.

Notwithstanding these limitations, this paper highlights the need for clinicians to audit prescribing patterns and increases awareness of the medication costs in patient care. In addition, it contributes to the literature regarding the treatment of bipolar depression in South Africa, for which there is a marked paucity of literature. Further studies on cost-effective maintenance treatment of BD in South Africa are recommended to inform both patient care and the NEML.

## Conclusion

Quetiapine use was associated with a highly significant improvement in functioning in our sample of patients. However, it came at a 52% increase in medicine cost. Pre-quetiapine treatments, though, did not achieve an optimal level of functioning, and overall costs may be reduced by more rational prescribing habits. Simply creating awareness of the medicine costs of managing such patients may improve resource utilisation and reduce inappropriate prescribing.

## References

[1] Nethathe G Cost awareness on the part of health professionals. S Afr Med J. 2015;105(8):618 10.7196/SAMJnew.7810 26543934

[2] World Health Organization WHO model list of essential medicines [homepage on the Internet]. 19th ed. Geneva: WHO; 2015 Available from: http://www.who.int/medicines/publications/essentialmedicines/en/

[3] Fekadu A, Hanlon C, Thornicroft G, et al Care for bipolar disorder in LMICs needs evidence from local settings. Lancet Psychiatry. 2015;2(9):772–773. 10.1016/S2215-0366(15)00222-9 26360884

[4] FDA US Seroquel XR FDA approval: Food and Drug Administration of the United States [homepage on the Internet]. Available from: http://www.accessdata.fda.gov/drugsatfda_docs/label/2011/022047s023s027lbl.pdf

[5] Parrish A, Blockman M Clinical excellence and the NICEties of value-based priority setting. S Afr Med J. 2008;98(10):758, 760–761. 19115747

[6] NDOH Standard treatment guidelines and essential medicine list for South Africa Hospital level adults. Pretoria, South Africa: National Department of Health; 2015.

[7] Colin F Bipolar disorder. S Afr J Psychiatry. 2013;19(3):164–171.

[8] Nierenberg AA, McElroy SL, Friedman ES, et al Bipolar CHOICE (Clinical Health Outcomes Initiative in Comparative Effectiveness): A pragmatic 6-month trial of lithium versus quetiapine for bipolar disorder. J Clin Psychiatry. 2016;77(1):90–99. 10.4088/JCP.14m09349 26845264

[9] Grunze H, Vieta E, Goodwin GM, et al The World Federation of Societies of Biological Psychiatry (WFSBP) guidelines for the biological treatment of bipolar disorders: Update 2010 on the treatment of acute bipolar depression. World J Biol Psychiatry. 2010;11(2):81–109. 10.3109/15622970903555881 20148751

[10] Grunze H, Vieta E, Goodwin GM, et al The World Federation of Societies of Biological Psychiatry (WFSBP) guidelines for the biological treatment of bipolar disorders: Update 2012 on the long-term treatment of bipolar disorder. World J Biol Psychiatry. 2013;14(3):154–219. 10.3109/15622975.2013.770551 23480132

[11] American Psychiatric Association DSM-5 Task Force Diagnostic and statistical manual of mental disorders: DSM-5. 5th ed. Washington, DC: American Psychiatric Association; 2013.

[12] Weissman MM, Wickramaratne P, Nomura Y, Warner V, Pilowsky D, Verdeli H Offspring of depressed parents: 20 years later. Am J Psychiatry. 2006;163(6):1001–1008. 10.1176/ajp.2006.163.6.1001 16741200

[13] Essential Drugs Programme Master procurement catalogue [homepage on the Internet]. Pretoria: Department of Health, South Africa; 2015 [cited 2015 Jun]. Available from: http://www.health.gov.za/index.php/component/phocadownload/category/196

[14] Asmal L, Flegar SJ, Wang J, Rummel-Kluge C, Komossa K, Leucht S Quetiapine versus other atypical antipsychotics for schizophrenia. Cochrane Database Syst Rev. 2013;11:CD006625 10.1002/14651858.cd006625.pub3 PMC11932229 24249315

[15] Statistics South Africa Census 2011 municipal fact sheet [homepage on the Internet]. Pretoria; 2012 [cited 2015 Jul]. Available from: http://www.statssa.gov.za/census/census_2011/census_products/Census_2011_Municipal_fact_sheet.pdf

[16] Robertson LJ, Szabo CP Community mental health services in Southern Gauteng, South Africa; an audit using data routinely collected by the District Health Information Systems. Submitted for publication 2016. 10.4102/sajpsychiatry.v23i0.1055 PMC6138200 30263192

[17] Aas IHM Collecting Information for Rating Global Assessment of Functioning (GAF): Sources of information and methods for information collection. Curr Psychiatry Rev. 2014;10(4):330–347. 10.2174/1573400509666140102000243 25598769 PMC4287015

[18] Pedersen G, Karterud S The symptom and function dimensions of the Global Assessment of Functioning (GAF) scale. Compr Psychiatry. 2012;53(3):292–298. 10.1016/j.comppsych.2011.04.007 21632038

[19] Song HR, Kwon Y-J, Bahk W-M, Woo YS, Lee H-B, Lee J, et al Current prescription pattern of maintenance treatments for bipolar patients in Korea: A focus on the transition from acute treatments. Psychiatr Clin Neurosci. 2015;70(1):42–50. 10.1111/pcn.12337 26243698

[20] Pollmann AS, Murphy AL, Bergman JC, Gardner DM Deprescribing benzodiazepines and Z-drugs in community-dwelling adults: A scoping review. BMC Pharmacol Toxicol. 2015;16:19 10.1186/s40360-015-0019-8 26141716 PMC4491204

[21] Bobo WV, Reilly-Harrington NA, Ketter TA, et al Effect of adjunctive benzodiazepines on clinical outcomes in lithium- or quetiapine-treated outpatients with bipolar I or II disorder: Results from the Bipolar CHOICE trial. J Affect Disord. 2014;161:30–35. 10.1016/j.jad.2014.02.046 24751304 PMC4113323

[22] Ekman M, Lindgren P, Miltenburger C, Meier G, Locklear JC, Chatterton ML Cost effectiveness of quetiapine in patients with acute bipolar depression and in maintenance treatment after an acute depressive episode. Pharmacoeconomics. 2012;30(6):513–530. 10.2165/11594930-000000000-00000 22591130

[23] Mitchell PB, Hadzi-Pavlovic D, Evoniuk G, Calabrese JR, Bowden CL A factor analytic study in bipolar depression, and response to lamotrigine. CNS Spectr. 2013;18(4):214–224. 10.1017/S1092852913000291 23702258

